# Supplementary material for: A geographic history of human genetic ancestry
Source: bioRxiv. 2024 Aug 19:2024.03.27.586858. Originally published 2024 Mar 29. Preprint. [Version 2] doi: 10.1101/2024.03.27.586858 (PMC10996620; doi:10.1101/2024.03.27.586858)
Supplement: Supplement 1 [file NIHPP2024.03.27.586858v2-supplement-1.pdf]

bioRxiv preprint doi: https://doi.org/10.1101/2024.03.27.586858; this version posted August 19, 2024. The copyright holder for this preprint (which was not certified by peer review) is the author/funder, who has granted bioRxiv a license to display the preprint in perpetuity. It is made available under aCC-BY 4.0 International license.

Supplementary Information

Appendix

A1. Overview

We first review generalized parsimony as it applies to a single gene tree (Sankoff, 1975; Sankoff and Rousseau, 1975). For each node u we maintain three cost functions. The *node cost* function $g_u(x)$ assigns a cost to each geographic state x (when u is in state x) that gives the minimum migration cost required to explain the geographic states of all sample nodes whose most recent common ancestor is u. If u is a sample node, we set $g_u(x) = 0$ if $x = x_u$ and $g_u(x) = \infty$ if $x \neq x_u$. When u is not a sample node, g_u is formed by the sum of the *stem cost* functions of its children.

The stem cost function for a node u is given by $h_u(x) = \min_z\left[\Delta_u(x,z) + g_u(z)\right]$. The function $\Delta_u(x,z)$ assigns a cost to the migration from state x to state z over the branch leading to node u. Here, we assume that Δ_u can be factored as: $\Delta_u(x,z) = \phi(\tau_u) * \Delta(x,z)$, where τ_u is the length of the branch leading to node u and Δ is a time-independent transition cost function. Typical choices for the time-dependent component are $\phi(\tau_u) = \frac{1}{\tau_u}$ or $\phi(\tau_u) = 1$, the latter making the whole cost function independent of branch lengths.

The node cost and stem cost functions can be computed in a single post-order tree traversal. A second pre-order tree traversal can then be used to compute the *final cost* functions for each node. The final cost $f_u(x)$ gives the minimum migration cost required to explain the geographic states of *all* sample nodes when u is in state x. When u is the root of the tree, $f_u(x) = g_u(x)$. Otherwise, $f_u(x) = \min_z\left[f_{\pi_u}(z) - h_u(z) + \Delta_u(z,x)\right] + g_u(x)$, where π_u represents the immediate ancestor of node u.

A2. Algorithm

Here we describe how we use the succinct tree sequence encoding (Kelleher et al., 2016) to efficiently compute node cost and stem cost functions for each local genealogy. Under the coalescent with recombination, nearby gene trees are highly correlated. Moving between adjacent local trees typically requires only a small number of subtree-prune-and-regraft (SPR) operations. By keeping an index of the edges involved in these SPR operations and the order in which they need to be applied, the succinct tree sequence encoding allows us to efficiently maintain the state of parsimony calculations as we iterate over the local genealogies.

A detailed description of the tree sequence data structure can be found in Kelleher et al. (2016). For our purposes, it suffices to know that relationships among all N nodes in the tree sequence are recorded in the edge table E. Each row in the edge table records an ancestor-descendant relationship, and the indices of nodes involved in that relationship can be accessed as $E[k]$.`parent` and $E[k]$.`child`. The relationship encoded by an edge applies to the half-open genomic interval between $E[k]$.`left` (inclusive) and $E[k]$.`right` (exclusive). As we move along the genome from left to right, the index vectors I and O give the insertion and removal order of the edges needed to build each local gene tree topology (recorded in the vector π). The following algorithm is based on the branch statistic algorithm that appears in Ralph et al. (2020) but differs in its treatment of sample weights and how they are propagated along a genealogy.

Algorithm P (generalized parsimony). Given a set of georeferenced samples related by a tree sequence with length L, compute the genome-wide average final cost function $F_u(x)$ and the genome-wide average migration rate statistic σ_P. The function $F_u(x)$ returns the average minimum migration cost required to explain the observed geographic states of all sample nodes when u is in state x, where the average is taken over all local genealogies (weighted by their genomic span) where node u appears. The statistic σ_P is the average per-branch migration cost in a most parsimonious migration history averaged over all local genealogies weighted by their genomic span.

S1. [Initialization.] For $0 \leq u < N$ set $\pi_u \leftarrow -1$, $s_u \leftarrow 0$, $g_u(x) \leftarrow 0$, $F_u(x) \leftarrow 0$. Then, if u is a sample node and $x \neq x_u$, set $g_u(x) \leftarrow \infty$ and $F_u(x) \leftarrow \infty$

Finally, set $j \leftarrow 0$, $k \leftarrow 0$, $\sigma_P \leftarrow 0$, $t_l \leftarrow 0$, $s_P \leftarrow 0$.

S2. [Terminate.] If $j = |E|$ terminate.

S3. [Edge removal loop.] If $k = |E|$ or $t_l \neq E[O_k]$.`right` go to S6.

S4. [Remove edge.] Set $u \leftarrow E[O_k]$.`parent`, $v \leftarrow E[O_k]$.`child`, $w \leftarrow \pi_u$, and $k \leftarrow k+1$. Then, if $w \neq -1$, set $g_w(x) \leftarrow g_w(x) - h_u(x)$. Finally, set $g_u(x) \leftarrow g_u(x) - h_v(x)$, $\pi_v = -1$, $v \leftarrow u$, $u \leftarrow w$.

S5. [Update node and stem costs.] While $u \neq -1$, set $w \leftarrow \pi_u$ and if $w \neq -1$ set $g_w(x) \leftarrow g_w(x) - h_u(x)$. Then set $h_v(x) \leftarrow \min_z\left[\Delta_v(x,z) + g_v(z)\right]$, $g_u(x) \leftarrow g_u(x) + h_v(x)$, $v \leftarrow u$, $u \leftarrow w$. Afterward, go to S3.

bioRxiv preprint doi: https://doi.org/10.1101/2024.03.27.586858; this version posted August 19, 2024. The copyright holder for this preprint (which was not certified by peer review) is the author/funder, who has granted bioRxiv a license to display the preprint in perpetuity. It is made available under aCC-BY 4.0 International license.

S6. [Edge insertion loop.] If $j = |E|$ or $t_l \neq E[I_j]$.left go to S9.

S7. [Insert edge.] Set $u \leftarrow E[I_j]$.parent, $v \leftarrow E[I_j]$.child, $w \leftarrow \pi_u$, and $j \leftarrow j+1$. Then, if $w \neq -1$, set $g_w(x) \leftarrow g_w(x) - h_u(x)$. Then set $h_v(x) \leftarrow \min_z \left[\Delta_v(x,z) + g_v(z)\right]$. Finally, set $g_u(x) \leftarrow g_u(x) + h_v(x)$, $\pi_v = u$, $v \leftarrow u$, $u \leftarrow w$.

S8. [Update node and stem costs.] While $u \neq -1$, set $w \leftarrow \pi_u$ and if $w \neq -1$ set $g_w(x) \leftarrow g_w(x) - h_u(x)$. Then set $h_v(x) \leftarrow \min_z \left[\Delta_v(x,z) + g_v(z)\right]$, $g_u(x) \leftarrow g_u(x) + h_v(x)$, $v \leftarrow u$, $u \leftarrow w$. Afterward, go to S6.

S9. [Genomic span of tree.] Set $t_r \leftarrow L$. If $j < |E|$ set $t_r \leftarrow \min\left(t_r, E[I_j].\texttt{left}\right)$. Then, if $k < |E|$ set $t_r \leftarrow \min\left(t_r, E[O_k].\texttt{right}\right)$. Set $s \leftarrow t_r - t_l$.

S10. [Update average migration costs.] Set $\sigma \leftarrow 0$, $n \leftarrow 0$, $s_P \leftarrow s + s_P$. Then visit each node u in a pre-order traversal and set $s_u \leftarrow s_u + s$. If $\pi_u = -1$ set $f_u(x) \leftarrow g_u(x)$ and set $\sigma \leftarrow \sigma + \min_z f_u(z)$; otherwise, set $f_u(x) \leftarrow \min_z \left[f_{\pi_u}(z) - h_u(z) + \Delta_u(z,x)\right] + g_u(x)$ and set $n \leftarrow n+1$. Then, set $F_u(x) \leftarrow F_u(x) + s \times \frac{f_u(x) - F_u(x)}{s_u}$.

S11. [Update average migration rate.] Set $\sigma_P \leftarrow \sigma_P + s \times \frac{\sigma/n - \sigma_P}{s_P}$.

S12. [Tree loop tail.] Set $t_l \leftarrow t_r$. Go to S2.

We begin in S1 by initializing the cost functions for each node to zero except in the case of sample nodes, for which we set the cost function to positive infinity for all states not equal to the observed state. We then set the parent of each node to -1 (signifying the null element) so that the initial state of the tree sequence is a forest of disconnected nodes. The average cost functions (denoted by the corresponding capital letters) are also initialized to 0 as these will be updated as we iterate over the tree sequence.

The meat of the algorithm occurs in steps S4 and S5 of the edge removal loop and in steps S7 and S8 of the edge insertion loop. Removal of an edge from node u to v will alter the node and stem cost functions along the path from u back to the root of the genealogy. Step S4 prepares for this by first subtracting the stem costs of u and v from the node costs of their respective parents. In step S5, we walk back along the path from u's parent to the root and recompute new stem costs and node costs given the updated node costs at the head of the path. Insertion of an edge from node u to v will similarly alter the node and stem cost functions along the path from u back to the root of the genealogy. Step S7 prepares for this by first substracting the stem cost of u from the node cost of its parent and then computing the stem cost of v and adding it to the node cost of u. In step S8, we walk back along the path from u's parent to the root as before and recompute new stem costs and node costs given the updated node costs at the head of the path.

Upon reaching step S9, we have finished constructing the tree together with its node cost and stem cost functions. We record the genomic span of the tree in the variable s, which will be the weight applied to the current tree in the weighted average. In S10, we perform a preorder traversal of the newly constructed tree and compute the final cost function for each node. At the same time, we increment the total weight s_u of each node by s and update the weighted averages $F_u(x)$ with the cost functions for the current tree. When we begin the traversal at the root(s) of the tree we also record the minimum migration cost σ needed to explain the sample distribution. We use this cost in S11, together with the number n of edges in the tree, to update the weighted average migration rate statistic σ_P.

Correctness of the algorithm requires that edges are removed in order of nondecreasing right genomic coordinate and decreasing time and that edges are inserted in order of nondecreasing left genomic coordinate and increasing time (time is measured backward from the present). We assume that the index vectors I and O are constructed to satisfy these conditions; these assumptions form the basis of the conditional checks in steps S3 and S6 for determining when to exit the edge removal and insertion loops.

Relation to existing work

Several existing approaches to geographic inference with tree sequences merit discussion in relation to our own work. Wohns et al. (2022) introduced a nonparametric approach that estimates ancestor locations by successively averaging the coordinates of sample locations in a postorder traversal of the ARG to their most recent common ancestor. The resulting estimates are local estimates in the sense that the inferred location of an ancestor depends only on the locations of samples that trace some portion of their ancestry to that ancestor and on the topology of the corresponding subset of the ARG. By contrast, our approach estimates the location of an ancestor using information from all samples. Because all samples share common ancestry at some time in the past, even those samples that are not direct descendants of an ancestor can be informative about that ancestor's location.

Osmond and Coop (2021) describe a likelihood method for locating genetic ancestors and estimating migration rates that is based on a model of branching Brownian motion. Their approach also uses information from all samples to

bioRxiv preprint doi: https://doi.org/10.1101/2024.03.27.586858; this version posted August 19, 2024. The copyright holder for this preprint (which was not certified by peer review) is the author/funder, who has granted bioRxiv a license to display the preprint in perpetuity. It is made available under aCC-BY 4.0 International license.

estimate ancestral locations and can optionally estimate separate migration rates for deep and shallow time horizons. Unlike our method, inference is carried out on a sample of widely spaced genealogies rather than on the full tree sequence. We note that when squared Euclidean distance weighted by inverse branch length is used as the transition cost function, the maximum parsimony reconstruction on each marginal genealogy has highest posterior probability under a Brownian motion dispersal process. In this sense, the genome-wide average reconstruction produced by our method can be viewed as a weighted average of posterior modes. Deraje et al. (2024) recently extended the model of branching Brownian motion to work with the full ARG rather than a sample of local gene trees.

Bibliography

Deraje, P., Kitchens, J., Coop, G., and Osmond, M. M. (2024). Inferring the geographic history of recombinant lineages using the full ancestral recombination graph. *bioRxiv*. doi: 10.1101/2024.04.10.588900.

Kelleher, J., Etheridge, A. M., and McVean, G. (2016). Efficient coalescent simulation and genealogical analysis for large sample sizes. *PLOS Computational Biology*, 12(5):1–22. doi: 10.1371/journal.pcbi.1004842.

Osmond, M. M. and Coop, G. (2021). Estimating dispersal rates and locating genetic ancestors with genome-wide genealogies. *bioRxiv*. doi: 10.1101/2021.07.13.452277.

Ralph, P., Thornton, K., and Kelleher, J. (2020). Efficiently Summarizing Relationships in Large Samples: A General Duality Between Statistics of Genealogies and Genomes. *Genetics*, 215(3): 779–797. doi: 10.1534/genetics.120.303253.

Sankoff, D. (1975). Minimal mutation trees of sequences. *SIAM Journal on Applied Mathematics*, 28(1):35–42.

Sankoff, D. and Rousseau, P. (1975). Locating the vertices of a steiner tree in an arbitrary metric space. *Mathematical Programming*, 9:240–246.

Wohns, A. W., Wong, Y., Jeffery, B., Akbari, A., Mallick, S., Pinhasi, R., Patterson, N., Reich, D., Kelleher, J., and McVean, G. (2022). A unified genealogy of modern and ancient genomes. *Science*, 375(6583):eabi8264. doi: 10.1126/science.abi8264.

bioRxiv preprint doi: https://doi.org/10.1101/2024.03.27.586858; this version posted August 19, 2024. The copyright holder for this preprint (which was not certified by peer review) is the author/funder, who has granted bioRxiv a license to display the preprint in perpetuity. It is made available under aCC-BY 4.0 International license.

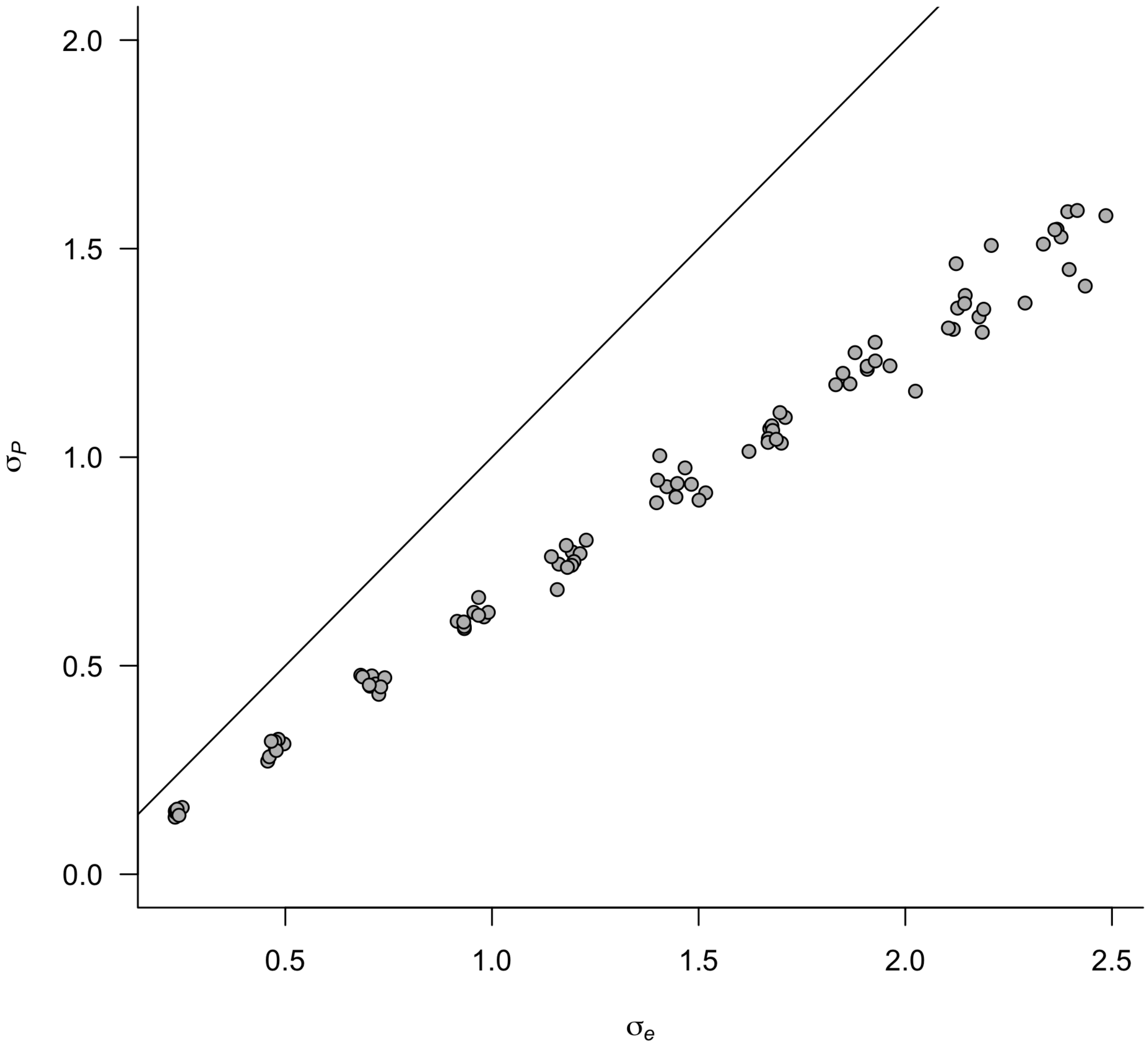

Figure S1. Migration rate estimates for a Gaussian dispersal kernel. Each point represents a single simulation generated under Gaussian dispersal with effective migration rate given on the x-axis and a parsimonious genome-wide estimate of that rate on the y-axis. The inset line shows a 1:1 relationship.

bioRxiv preprint doi: https://doi.org/10.1101/2024.03.27.586858; this version posted August 19, 2024. The copyright holder for this preprint (which was not certified by peer review) is the author/funder, who has granted bioRxiv a license to display the preprint in perpetuity. It is made available under aCC-BY 4.0 International license.

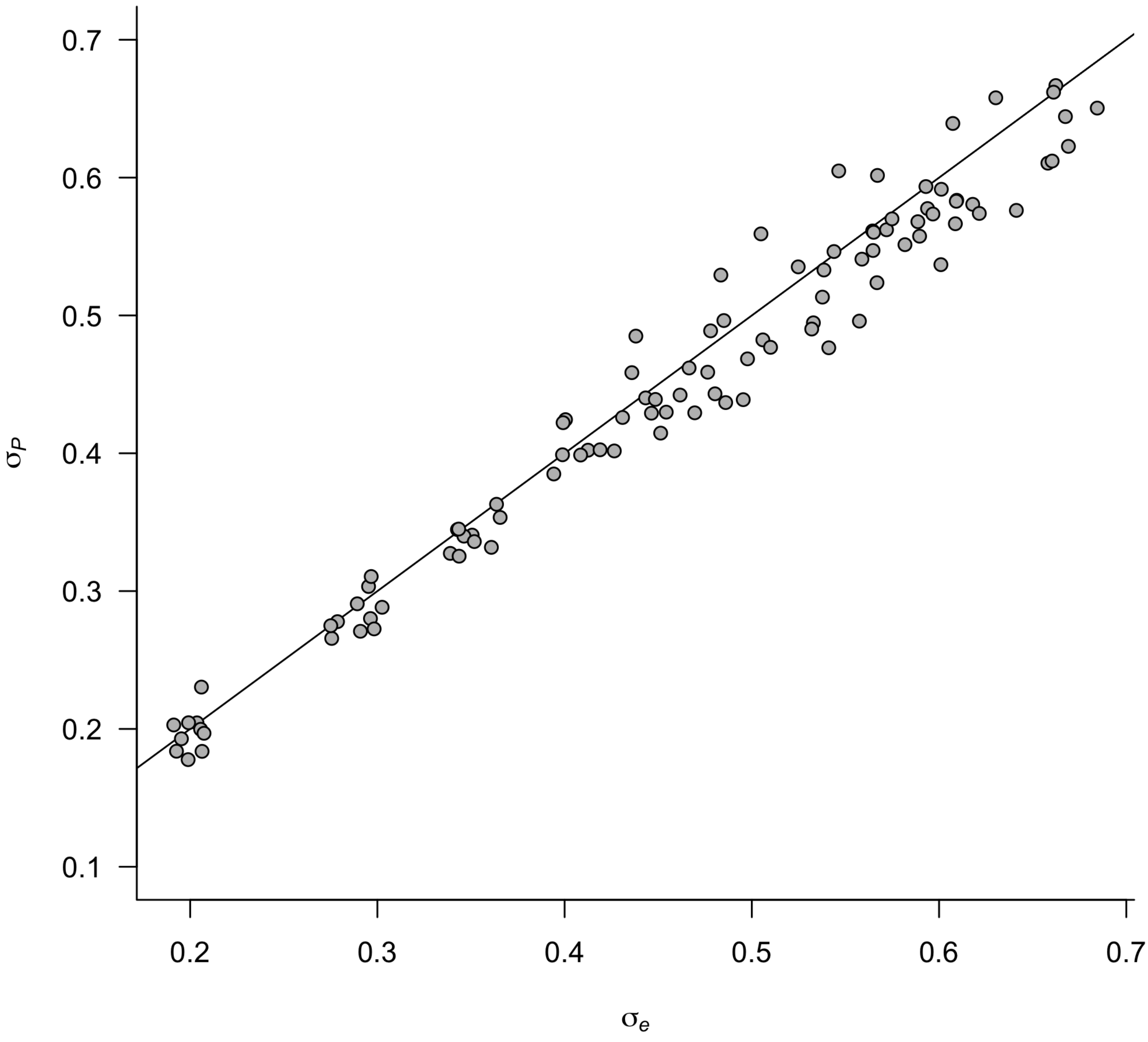

Figure S2. Migration rate estimates for a Laplace dispersal kernel. Each point represents a single simulation generated under Laplace dispersal with effective migration rate given on the x-axis and a parsimonious genome-wide estimate of that rate on the y-axis. The inset line shows a 1:1 relationship.

bioRxiv preprint doi: https://doi.org/10.1101/2024.03.27.586858; this version posted August 19, 2024. The copyright holder for this preprint (which was not certified by peer review) is the author/funder, who has granted bioRxiv a license to display the preprint in perpetuity. It is made available under aCC-BY 4.0 International license.

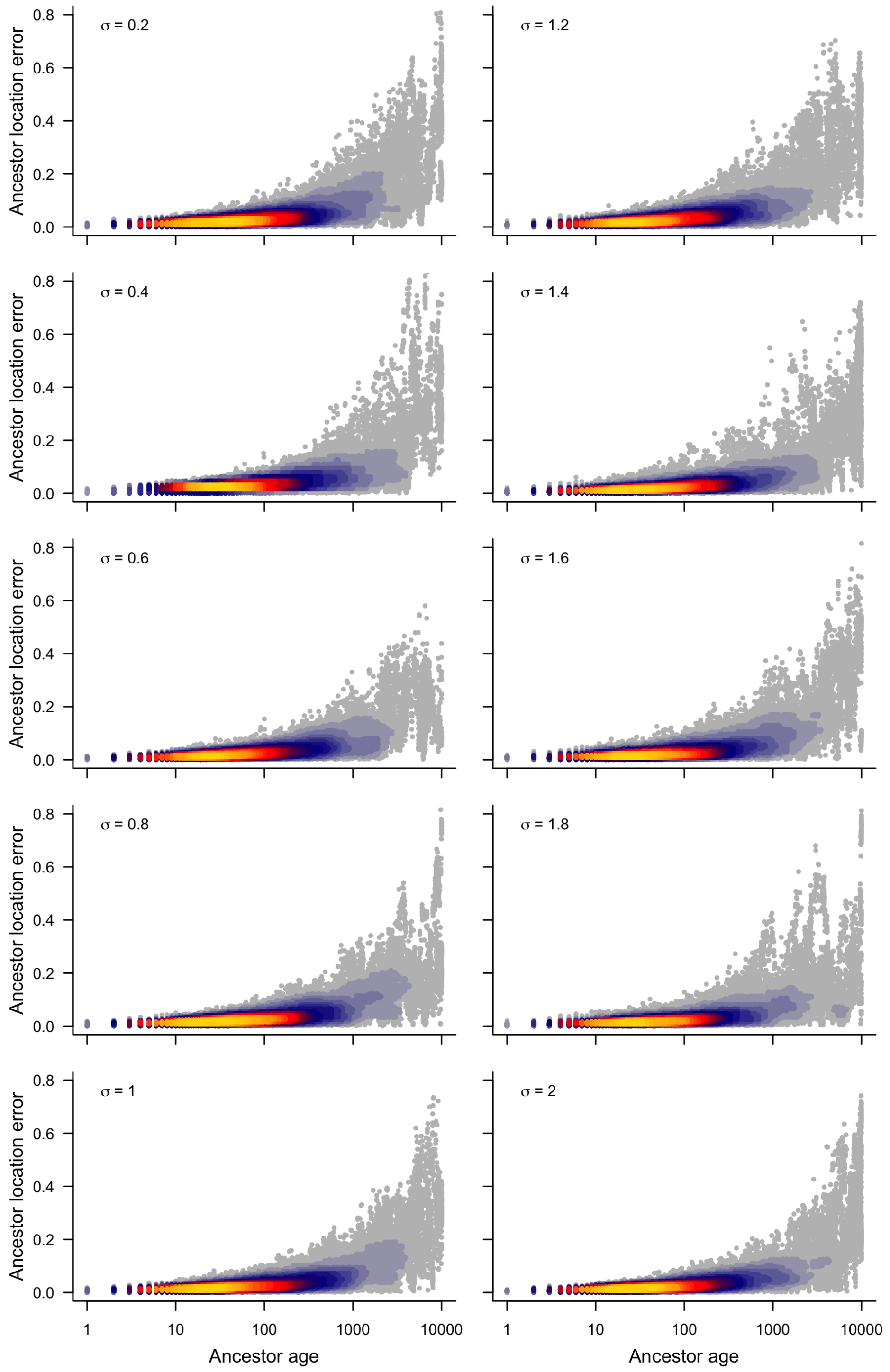

Figure S3. Ancestor location error for a Gaussian dispersal kernel. Each point represents a single genetic ancestor. Ancestor location error is measured as the distance between the estimated and the true location divided by the greatest distance separating any pair of samples. Warm colors signify a greater density of points.

bioRxiv preprint doi: https://doi.org/10.1101/2024.03.27.586858; this version posted August 19, 2024. The copyright holder for this preprint (which was not certified by peer review) is the author/funder, who has granted bioRxiv a license to display the preprint in perpetuity. It is made available under aCC-BY 4.0 International license.

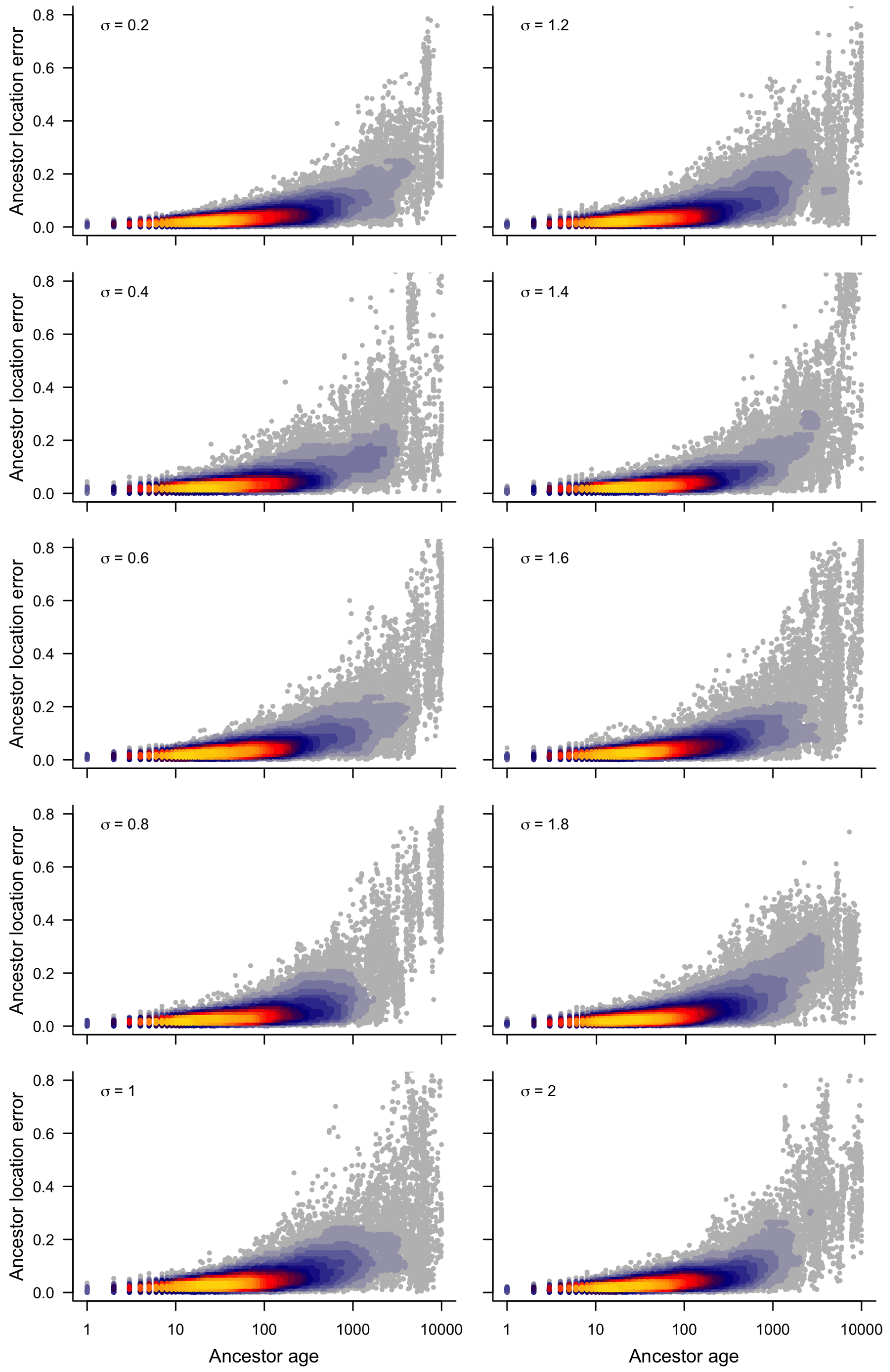

Figure S4. Ancestor location error for a Laplace dispersal kernel. Each point represents a single genetic ancestor. Ancestor location error is measured as the distance between the estimated and the true location divided by the greatest distance separating any pair of samples. Warm colors signify a greater density of points.
